# Supplementary material for: Circum-Antarctic bottom water formation mediated by tides and topographic waves
Source: Nat Commun. 2024 Mar 6;15:2049. doi: 10.1038/s41467-024-46086-1 (PMC10918180; doi:10.1038/s41467-024-46086-1)
Supplement: Supplementary file 1 — Supplementary Information [file 41467_2024_46086_MOESM1_ESM.pdf]

Circum-Antarctic bottom water formation mediated by tides and topographic waves

Xianxian Han[1,3], Andrew L. Stewart[2], Dake Chen[1,3,4*], Markus Janout[5], Xiaohui Liu[4], Zhaomin Wang[1], Arnold L. Gordon[6]

1 Southern Marine Science and Engineering Guangdong Laboratory (Zhuhai), Zhuhai, China

2 Department of Atmospheric and Oceanic Sciences, University of California, Los Angeles, California, USA

3 School of Atmospheric Sciences, Sun Yat-sen University, Zhuhai, China

4 State Key Laboratory of Satellite Ocean Environment Dynamics, Second Institute of Oceanography, Ministry of Natural Resources, China

5 Alfred Wegener Institute, Helmholtz Centre for Polar and Marine Research, Bremerhaven, Germany

6 Lamont Doherty Earth Observatory, Columbia University, Palisades, NY, USA

Corresponding author: Dake Chen, dchen@sio.org.cn

This PDF contains 10 supplementary figures and 1 supplementary table.

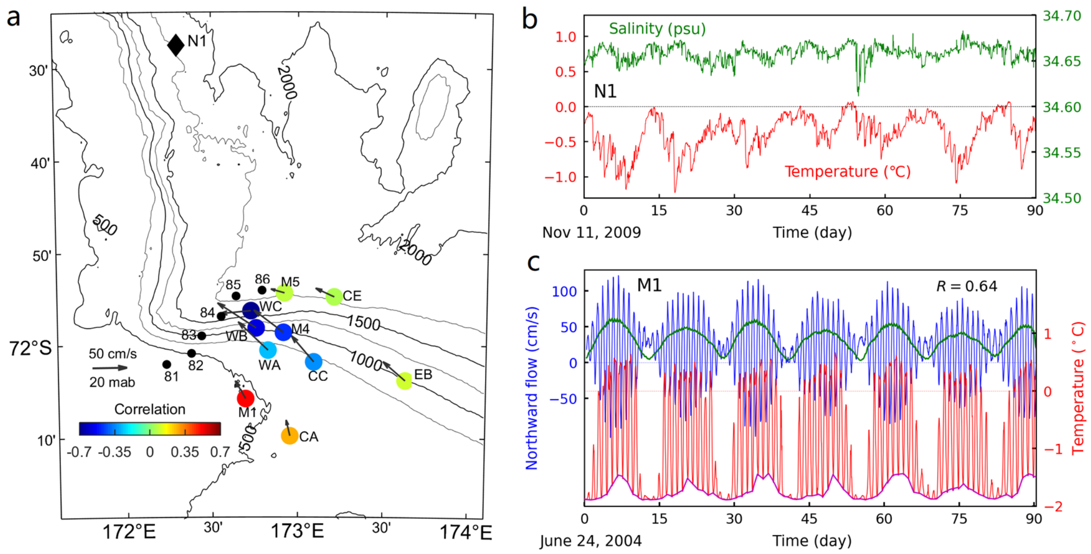

Supplementary Figure 1 | Observations in the Ross Sea overflow. (a) The colored dots indicate all available moorings on the continental slope at ~20 meters above bottom (mab), and the color shading denotes the correlation between the tidal flow strength and the daily minimum temperature for the whole observational period (~ 1 year) (see e.g. the time series from mooring WC in Fig. 1). The arrows indicate flow during 3 days neap tides, suggesting that there is still strong overflow during neap tides. (b) Time series of temperature and salinity from the downstream mooring N1 (25 mab). The temperature exhibits significant spring-neap tidal cycles. (c) Time series of temperature and velocity for mooring M1 at the shelf break region. The thick green line indicates the fitted daily tidal flow strength (see Methods), and the thick magenta line indiates the daily minimum temperature. The correlation between the green and magenta lines is 0.64.

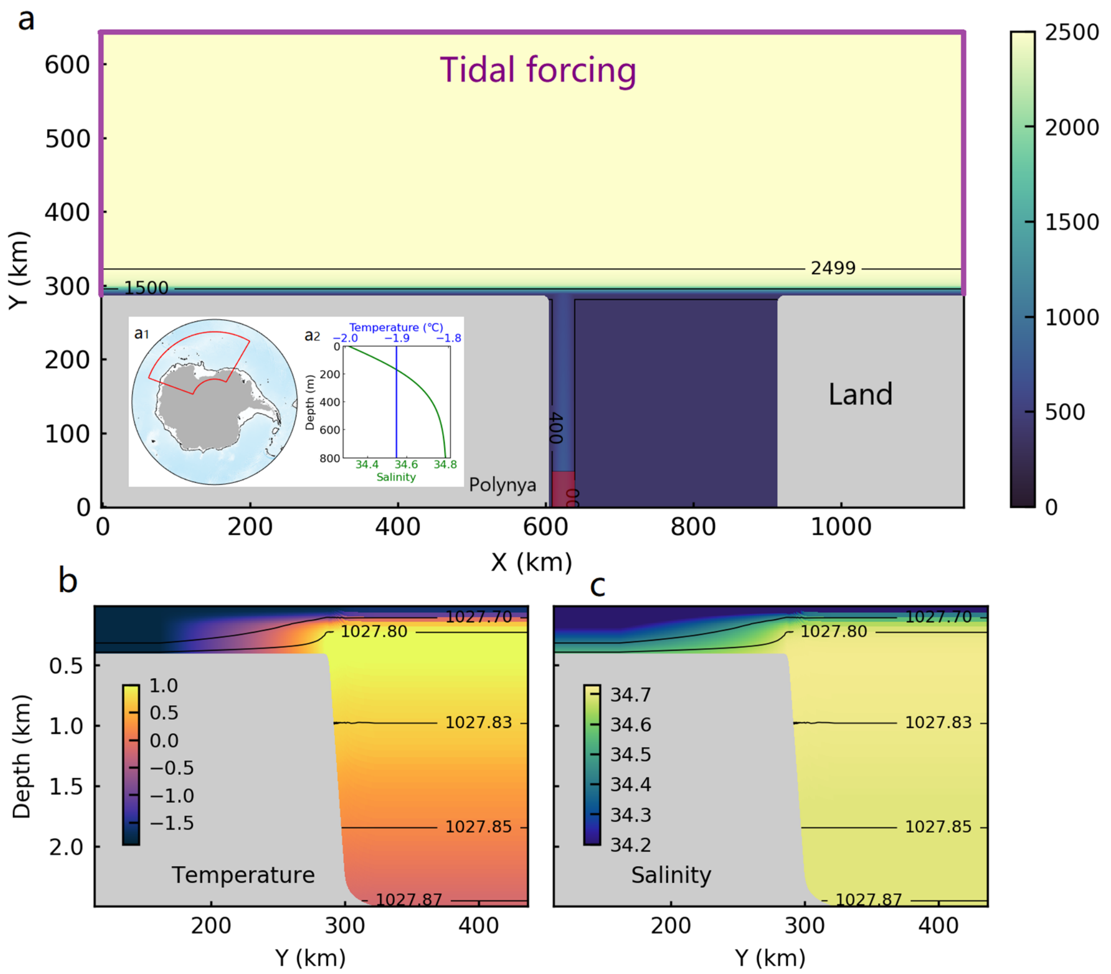

Supplementary Figure 2 | Model setup. (a) Topography of the model setup. Tidal motions are forced via imposed sea surface height fluctuations at the open boundaries, indicated by the thick purple line. The amplitude and phase of the tidal forcing is prescribed by extracting the K1 component (TPXO7) of the tidal sea surface height fluctuations around the Ross Sea, indicated by the red sector in inset a1. The semitransparent red shading at the south boundary of the trough indicates the existence of the dense shelf water formation site, which crudely approximates the effect of a coastal polynya: we restore the temperature and salinity toward the vertical profiles shown by inset a2. (b-c) Depth/latitude cross-section showing the zonally-invariant initial potential temperature and salinity in the model domain. Black contours indicate potential density referenced to surface.

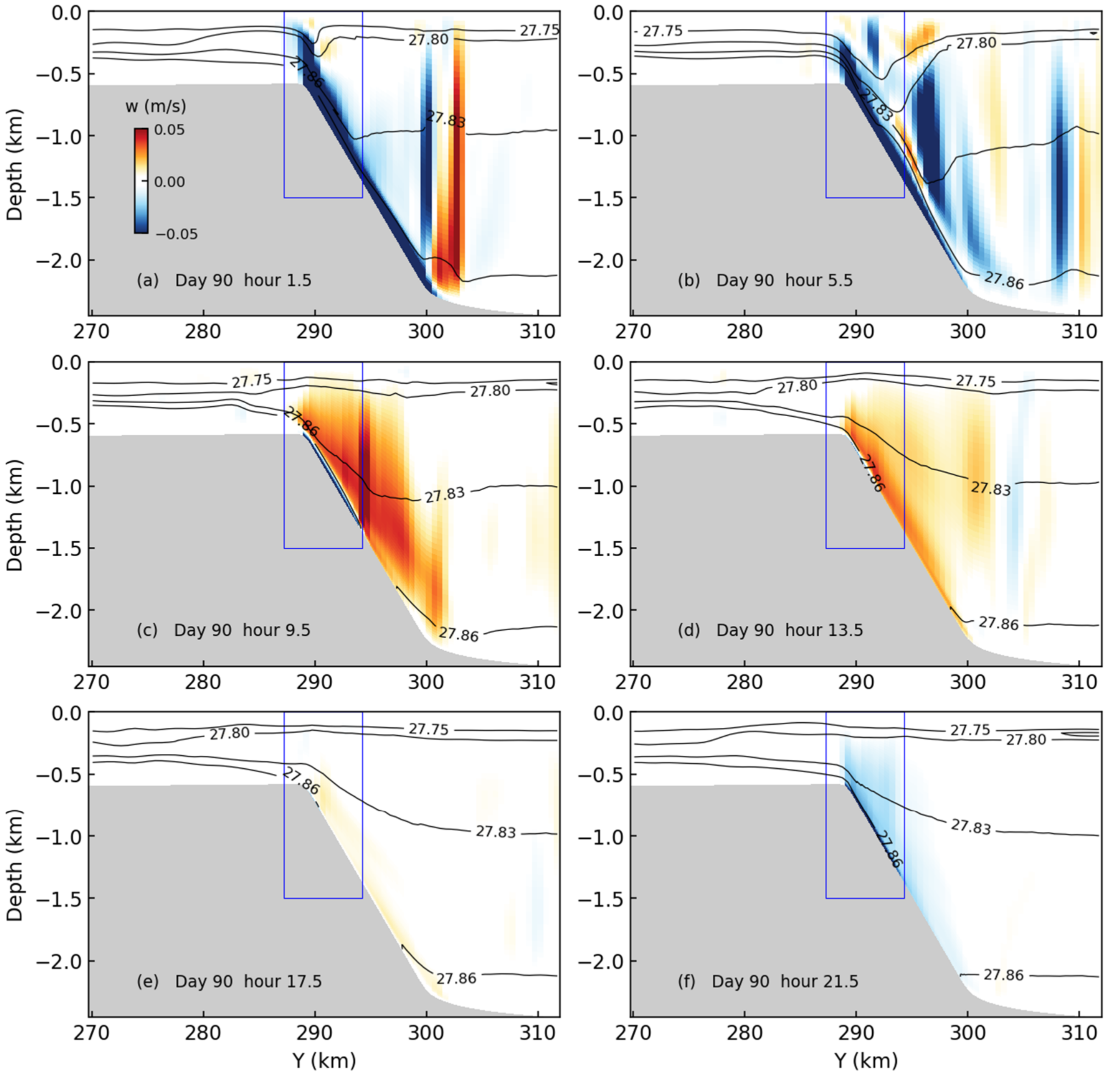

Supplementary Figure 3 | Vertical velocity variations over one tidal cycle. A sequence of cross-slope sections showing the variations of vertical velocity (color shading, with positive value indicating upward) and potential density contours (with 1000 kg/m³ subtracted), with interval of 4 hours. The blue rectangles indicate the location of tidal induced V-shaped front.

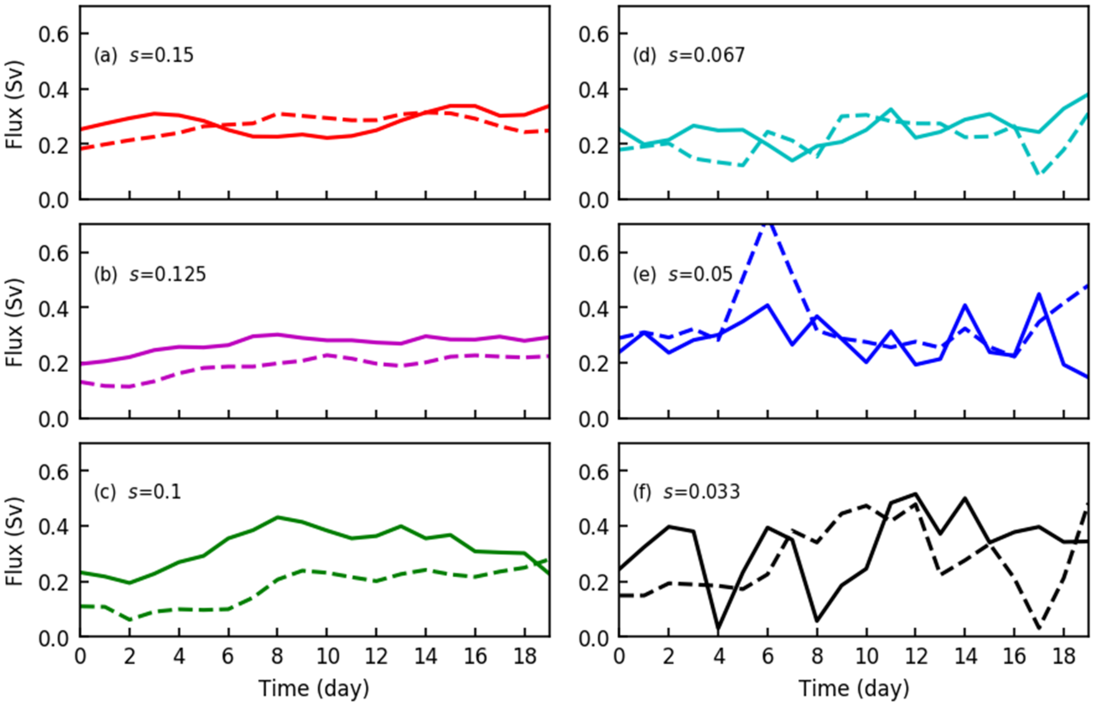

Supplementary Figure 4 | Overflow flux. Time series over the last 20 days of simulation time for 6 experiments with different slope inclines, showing daily-averaged overflow volume fluxes across continental shelf break. The solid and dashed lines indicate cases without and with tidal forcing, respectively. We define the overflow as all fluid with potential density (referenced to surface) larger than 1027.86 kg/m³.

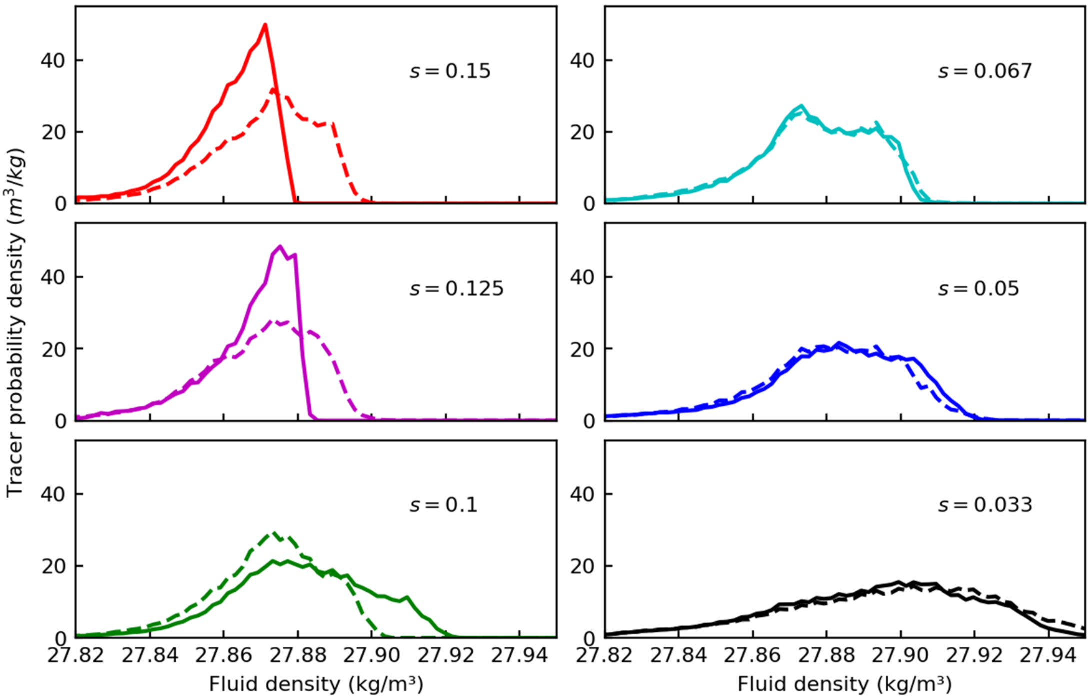

Supplementary Figure 5 | Probability density of volume-integrated tracer mass versus fluid potential density. Probability density of six different slopes experiments, with (dashed line) and without (solid line) tidal forcing.

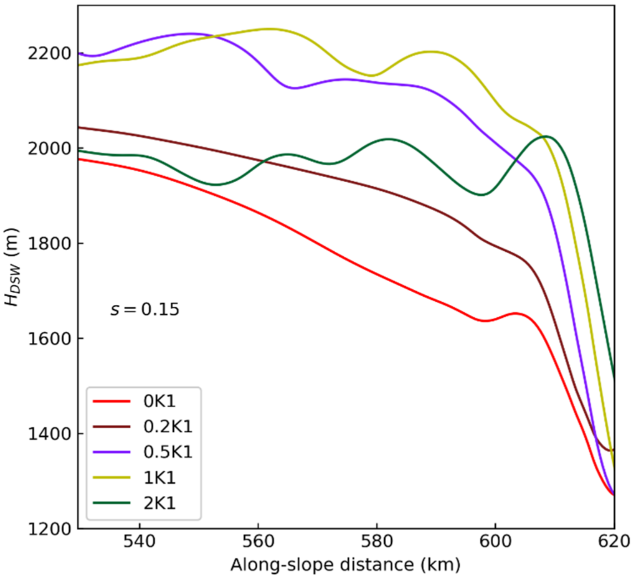

Supplementary Figure 6 | Descent pathway of Dense shelf water (DSW). Isobath corresponding to the DSW tracer center of mass, as a function of along-slope distance downstream of the trough. Here we vary tidal forcing strengths with constant slope steepness ($s = 0.15$).

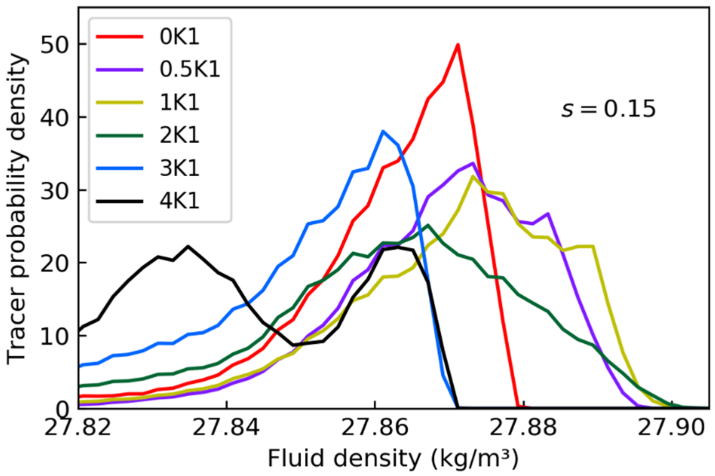

Supplementary Figure 7 | The impacts of tidal flow strength on Fluid density. Probability density of volume-integrated tracer mass versus fluid potential density for experiments with different tidal forcing but constant slope steepness ($s = 0.15$).

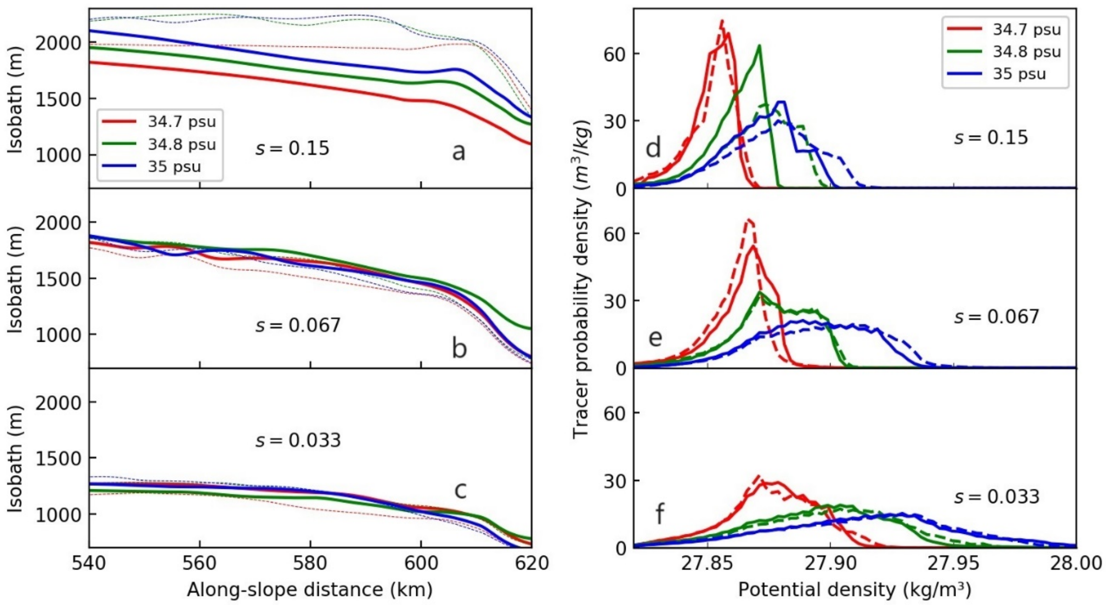

Supplementary Figure 8 | The impact of the density difference between dense shelf water (DSW) and surrounding waters on overflows. We vary the DSW density by restoring the salinities on the continental shelf to different maximum values, with a constant potential temperature of -1.9°C. The red, green and blue curves correspond to the DSW with maximum salinities of 34.7, 34.8 and 35 psu respectively. The solid thick curves indicate the simulations without tidal forcing, while the dashed curves indicate the corresponding simulations that include tidal forcing. (a-c) Isobath corresponding to the DSW tracer center of mass, as a function of along-slope distance downstream of the trough. The top to bottom panels correspond to steep (s=0.15), moderate (s=0.067) and small (s=0.033) slopes, respectively. (d-f) Probability density function of tracer as a function of potential density, computed over the area downstream of the semi-transparent red shading shown in Fig. 4a.

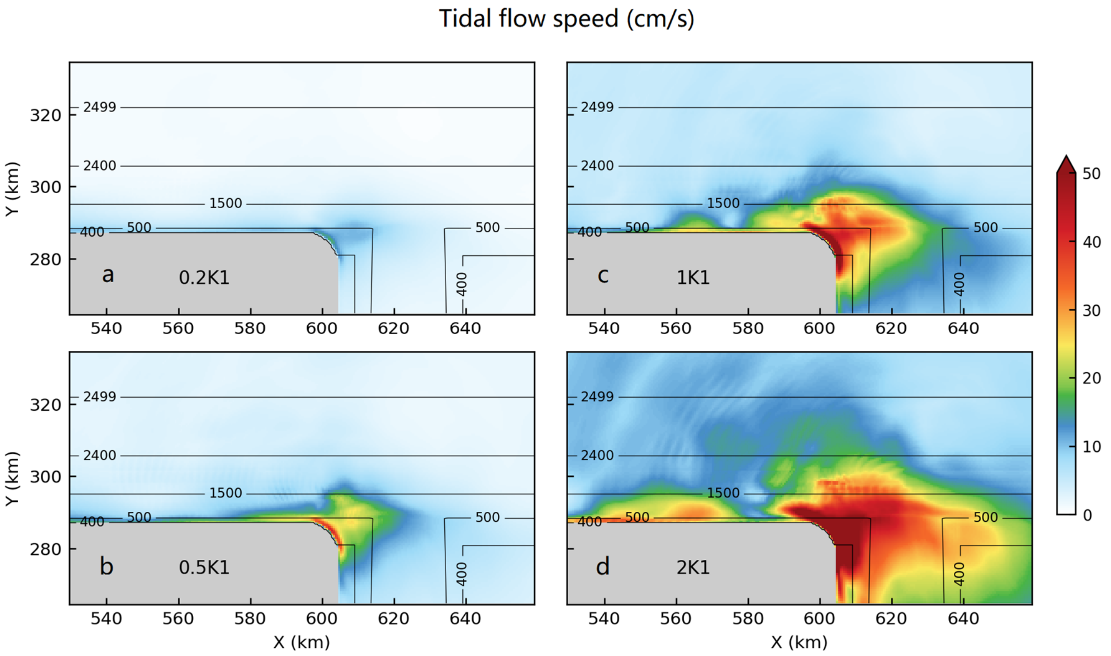

Supplementary Figure 9 | 10 day-averaged daily maximum tidal flow speed averaged over upper 200 m of the water column (see Methods). In all cases the tidal forcing uses the same variation of the phases along the boundary, based on the K1 tide from the Ross Sea, but with the amplitude uniformly multiplied by (a) 0.2, (b) 0.5, (c) 1 and (d) 2, respectively.

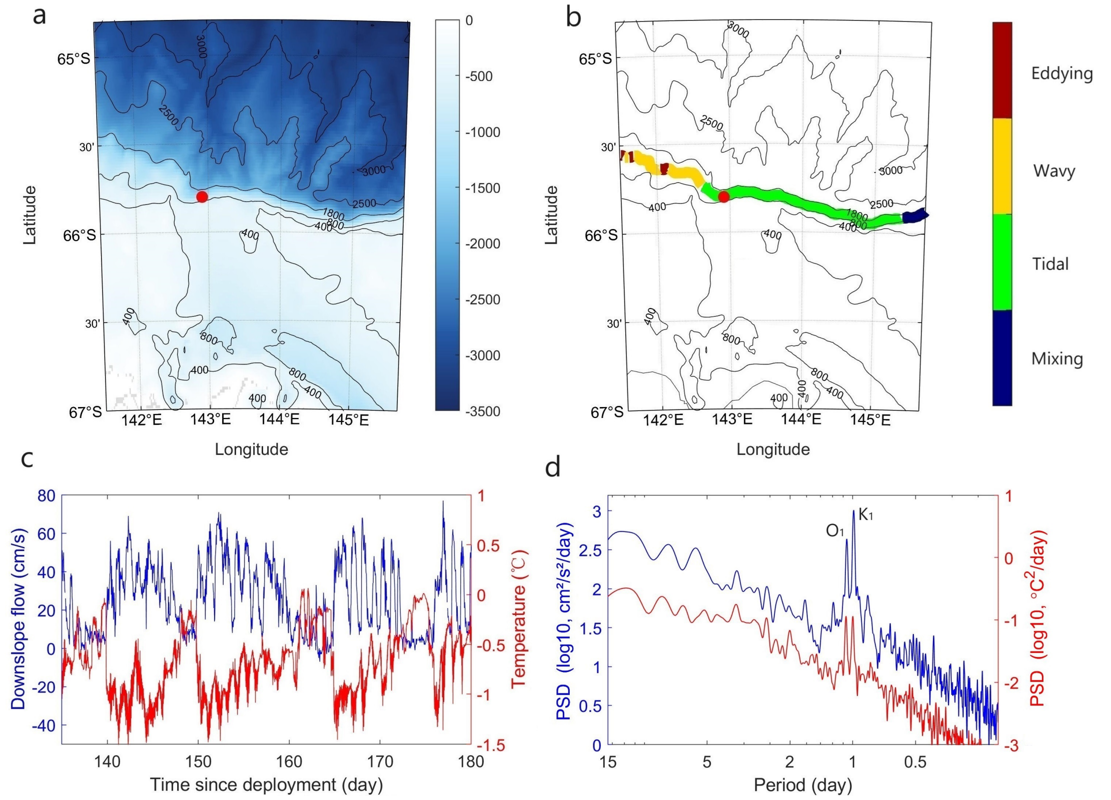

Supplementary Figure 10 | Adelie coast overflow observations. (a) Topography of the Adelie coastal region. The red dot indicates the mooring site, and the arrow denotes the export of DSW. (b) The estimated dynamical regimes around the Adelie coastal region. (c) Time series of northward velocity and temperature of selected 45 days. (d) Power spectral density (PSD) of northward velocity and temperature.

Supplementary Table 1 | Last 10 day-averaged overflow volume fluxes for experiments with varying bathymetric slopes, with or without tidal forcing. In general, the overflow volume fluxes of tidal experiments are smaller than those with no tidal forcing.

Slope\Flux (Sv)	No tide	Tide
S=0.15	0.29	0.28
S=0.125	0.28	0.21
S=0.1	0.34	0.23
S=0.067	0.29	0.24
S=0.05	0.26	0.31
S=0.033	0.39	0.31
